# Using Lower Limb Wearable Sensors to Identify Gait Modalities: A Machine-Learning-Based Approach

**DOI:** 10.3390/s23229241

**Published:** 2023-11-17

**Authors:** Liam David Hughes, Martin Bencsik, Maria Bisele, Cleveland Thomas Barnett

**Affiliations:** School of Science and Technology, Nottingham Trent University, Nottingham NG11 8NS, UK; liam.hughes@ntu.ac.uk (L.D.H.); martin.bencsik@ntu.ac.uk (M.B.);

**Keywords:** machine learning, accelerometer, wearable sensors, gait, principal component analysis, discriminant function analysis

## Abstract

Real-world gait analysis can aid in clinical assessments and influence related interventions, free from the restrictions of a laboratory setting. Using individual accelerometers, we aimed to use a simple machine learning method to quantify the performance of the discrimination between three self-selected cyclical locomotion types using accelerometers placed at frequently referenced attachment locations. Thirty-five participants walked along a 10 m walkway at three different speeds. Triaxial accelerometers were attached to the sacrum, thighs and shanks. Slabs of magnitude, three-second-long accelerometer data were transformed into two-dimensional Fourier spectra. Principal component analysis was undertaken for data reduction and feature selection, followed by discriminant function analysis for classification. Accuracy was quantified by calculating scalar accounting for the distances between the three centroids and the scatter of each category’s cloud. The algorithm could successfully discriminate between gait modalities with 91% accuracy at the sacrum, 90% at the shanks and 87% at the thighs. Modalities were discriminated with high accuracy in all three sensor locations, where the most accurate location was the sacrum. Future research will focus on optimising the data processing of information from sensor locations that are advantageous for practical reasons, e.g., shank for prosthetic and orthotic devices.

## 1. Introduction

A person’s gait can be a biomarker of global health status and aids in diagnosing pathologies and pathological progression [1,2,3,4,5,6]. The ability to vary gait speed can characterise functional impairment and predict the risk of falling [7,8,9,10]. Before recent developments in wearable sensor technology, gait analyses were traditionally conducted using motion capture technology in a laboratory [11]. 

Wearable sensors enable real-world gait assessment and are a more ecologically valid tool for characterising potential gait impairments. Developments in wearable technology and machine learning algorithms have facilitated a shift in gait evaluation into real-world scenarios, successfully identifying cyclical activities, e.g., running [12,13,14], negotiating stairs [15,16], and walking over differing terrains [13].

Wearable sensors can be attached to different locations on the human body. Choosing appropriate sites for attachment requires careful consideration. Sensors must be placed in a comfortable and realistic area for the user to wear that will not be affected by undesirable external factors, such as clothing articles or the knocking of body parts. Previous studies have explored attaching wearable sensors at the chest [17,18], wrist [12], hip [19,20], waist [21,22,23], thigh [4,24], shank [4] and foot [5,15,25]. The lower back is a frequently chosen attachment sites for collecting acceleration signals [1,26,27,28]. Attachment sites at the bottom of the back can produce success rates higher than 87% when classifying Parkinson’s disease [1], 94% when predicting age differences between participants [27] and 97% when predicting participants running speeds [26]. This location’s high success is likely due to the centre of mass dynamics at the lower back being the most representative of whole-body movement. Combining sensor data in a multi-sensor approach is also possible: placing several sensors on the body to combine raw acceleration or gyroscopic signals can, however, provide enhanced information on activity type and duration [6,29,30,31,32,33,34]. Using too many sensors can cause issues with participant engagement, particularly during a longer-term assessment, whether due to adhesion discomfort, faulty sensors, or general aesthetics. The sensor location makes for a sensitive consideration in pathological populations, for example, where recommended attachment locations may be in irritable or sensitive body parts. Ideally, the detection of gait modalities using wearable technology needs to use the fewest number of sensors attached at locations that will not impede the wearer’s comfort. If sensors located at the lower extremity (e.g., shanks) can accurately identify and discriminate gait, sensors can be attached to or integrated into external devices, such as orthoses or prosthetics, which would likely boost wearer conformity when collecting long-term data.

Previous research [15] has used inertial measurement units (IMUs), which can additionally incorporate gyroscopic information with raw acceleration data [18,22]. Gait speed and stride position have been estimated from IMUs located in footwear [35]; however, the utilisation of IMUs presents potential challenges for various reasons. IMUs provide more complex data due to their ability to measure not only linear acceleration (unlike accelerometers) but also angular velocity and orientation, providing additional data processing steps. Moreover, they are equipped with more substantial and weighty hardware, which could negatively impact long-term user adherence. Consequently, opting for a single accelerometer can provide clinicians and researchers a pathway for gait evaluation that enables longer recordings, entails lighter computational demands, and potentially enhances user compliance with device wear.

Previous research reports the success rate of different machine-learning-based algorithms using wearable sensors to identify various gait activities and modalities [15,18,21,22,31,32]. Although some of this research reported success rates above 99% [15], no study has quantitatively explored the ability of the algorithm to discriminate between gait modalities using a single lower extremity accelerometer. Multiple machine learning models can be used for activity recognition; models must be selected based on factors such as size, dimensionality, and, in particular, what goals are required from a dataset. Algorithms such as support vector machines (SVMs), hidden Markov models, and neural networks have reported success rates of 80–95% in activity recognition, including identifying terrains [27], falls [31], gait types [13,14] and other activities of daily living [36,37]. Although the success rate is one of the more critical conclusions, the outcome is binary, unable to provide the quality of a sensor’s ability to discriminate more than two gait modalities. Quantifying discrimination quality allows clinicians and researchers to make an informed decision on the best sensor attachment location. It also opens up the scope to modify and optimise algorithm performance, potentially allowing sites previously considered as ‘poor’ attachment locations to provide better performance outcomes and, consequently, better information for different treatment outcomes.

Using a single accelerometer potentially located on the lower limb to identify different gait modalities would allow for further developments in integration into an assistive device. Accelerometers are lightweight in design and are able to take long recordings for long-term assessments. Quantifying the success of how well the accelerometer can detect what gait modalities a person is executing would allow parameters in the algorithm to be optimised for further activity modalities to be identified in the future. The present study therefore aimed to quantify the performance of a simple machine learning algorithm in the discrimination of three gait modalities: self-selected slow, self-selected normal, and self-selected fast walking, using data from a single wearable sensor, attached to a range of commonly used physical attachment sites.

## 2. Methods

### 2.1. Participants

All recruited participants (N = 35, 19 ♂ 10 ♀, 27.4 ± 26.5 years, 1.74 ± 0.8 m, 71.5 ± 11.3 kg) were 18 and older and had no current musculoskeletal injury and no unresolved cardiovascular disease. Participants arrived wearing either shorts or skin-tight lycra leggings and trainers. The study was conducted in accordance with the Declaration of Helsinki, and approved by the Institutional Human Ethics Committee of Nottingham Trent University (protocol code 595 28 October 2020).

### 2.2. Study Design

**Figure 1:**
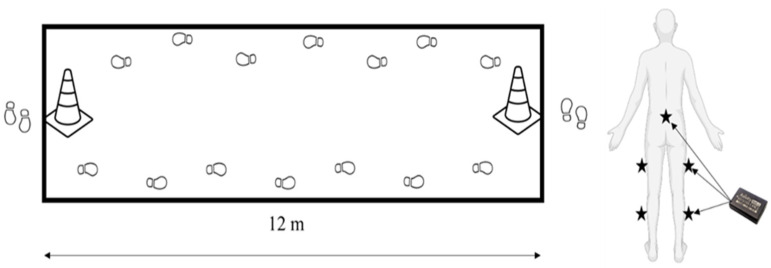
Diagram showing the experimental set-up and sensor attachment locations on the participants.

Participants completed 3 × 120 m flat overground (12 m walkway) walking trials under three experimental walking conditions (Figure 1), being instructed to walk at their perceived slow, normal (faster than slow) and fast (faster than normal) paces. Walking conditions were randomised between participants using a random number generator and separated by one-minute periods of quiet standing.

### 2.3. Instrumentation

Experimenters fitted participants with five independent triaxial accelerometers (Axivity AX6, York, UK) (100 Hz sampling rate, 8 g maximum acceleration). Sensors were adhered to the skin with double-sided adhesive tape at the sacrum and roughly the midpoint of the lateral thigh and lateral shank for both the right and left leg and were configured to log accelerations on three orthogonal planes (X, Y, Z).

### 2.4. Machine Learning Algorithm

#### 2.4.1. Pre-Processing

The magnitude of the acceleration vector for the time course of the raw acceleration signal was used. Linear interpolation was applied to synchronise each sensor to the same timestamp as sensors exhibited a slight drift in sampling frequency when preset at 100 Hz.

**Figure 2:**
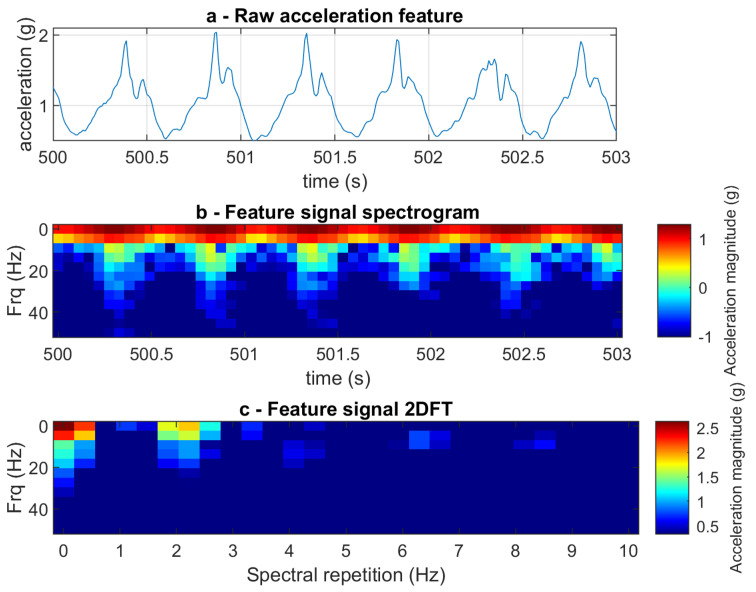
(**a**) Time course of a raw acceleration signal during a fast walking condition, showing six peaks in acceleration amplitude across the three-second period. (**b**) Spectrogram of the acceleration signal during a fast walking condition highlights six periods of large frequency bandwidth over three seconds. (**c**) Two-dimensional Fourier transform image of the fast walking, highlighting the spectrum of a repeated signal around two times per second.

**Figure 3:**
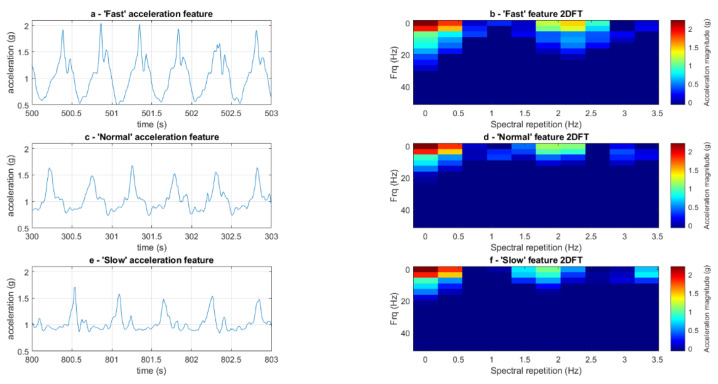
(**a**) Time course of a raw acceleration signal during a fast walking condition showing six periods of peak amplitudes over three seconds. (**b**) A two–dimensional Fourier transform (2DFT) image of the fast walking waveform, highlighting a repeating waveform occurring more than two times per second. (**c**) Same as ‘a’ for normal walking, showing six periods of peak amplitudes over three seconds. (**d**) Same as ‘b’ for normal walking, with a decreased observed frequency of the spectral repetition highlighting a repeated waveform occurring roughly two times per second. (**e**) Same as ‘a’ for slow walking, showing five periods of peak amplitudes over three seconds. (**f**) Same as ‘b’ for slow walking, with a decreased observed frequency of the spectral repetition highlighting a repeated waveform occurring, highlighting a repeating waveform occurring under two times per second.

**Figure 4:**
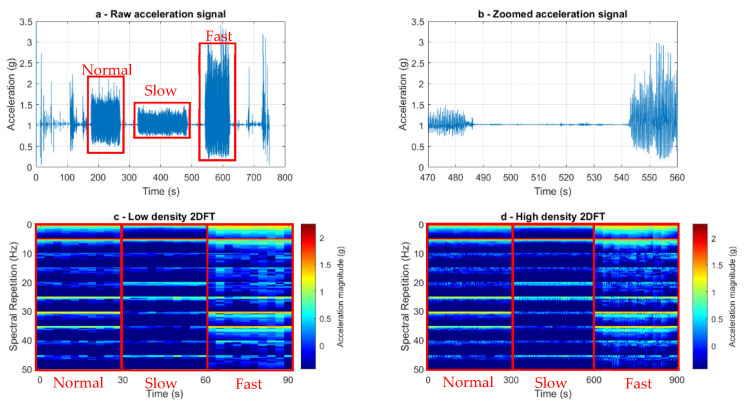
(**a**) Raw acceleration–time curves for one individual’s trial for sensors at the sacrum. The superimposed rectangular selections show three experimental conditions: normal, slow and fast walking. (**b**) A zoomed-in sacrum acceleration–time curve for fast walking. This shows the ten bouts of straight-line walking and the deceleration phases where the participant turned. These turns were dismissed from the analysis. (**c**) A stacked series of two-dimensional Fourier transform images displaying three experimental conditions. The superimposed rectangular selections show three experimental conditions: normal, slow and fast walking. Ten two-dimensional Fourier transform images were stacked per condition, which are three seconds in length, resulting in 10 spectra containing information on a three-second signal per experimental condition. Dark red indicates a higher acceleration magnitude, and dark blue indicates a lower magnitude. (**d**) A higher density series of two-dimensional Fourier transform images highlighting three separate experimental conditions. The superimposed rectangular selections show three experimental conditions: normal, slow and fast walking. Initial two-dimensional Fourier transform spectra are scanned temporally in time increments of 0.1 s over a period of one second, providing spectra for each experimental condition (100 × 3 s spectra per experimental condition).

Two-dimensional Fourier transform analysis (2DFT) [38] was used to analyse the acceleration signals during the experimental conditions (Figure 2). Signal features were selected from the acceleration signal from ten 3 s long known periods of walking per experimental condition (ten normal walking, ten slow walking, ten fast walking). A feature length of three seconds was selected as this is long enough to encompass at least one gait cycle, irrespective of gait speed. A temporal resolution of 0.3 s was chosen, dictating the time duration of the data on which one frequency spectrum is calculated. An overlapping factor of two was applied to the segmentation of the time-domain data, resulting in adjacent segments sharing 50% of their data points, enhancing the capture of transient frequency features during the 2DFT analysis (Figure 3). The 2DFT was applied to each selected three-second time slot, providing the features for analysis entirely within a frequency domain representation; this gives a feature that remains constant irrespective of the phasing of the analysis window. The 2DFT was then stretched into an array, representing acceleration amplitude at specific frequency components in the form of a two-dimensional spectrum. The individual 2DFT arrays from each segment were then stacked to create a larger 2DFT matrix (ten 2DFT per experimental condition). The 2DFT image revealed the fundamental frequency and associated harmonics observed from the acceleration signal, as well as providing a visualisation of their repeated spectral signatures within a signal [39]. Visual inspection of the series of 2DFTs allowed experimenters to check for any user error when selecting from known periods of walking, ensuring corners or periods of acceleration and deceleration were omitted from analysis (Figure 4).

A higher-density matrix of two-dimensional Fourier transform features was then also computed. Taking the initial, lower-temporal-density 2DFT signals, the 2DFT spectra were calculated in time increments of 0.1 s over a period of one second. The high-density scan provided 100 (10 features multiplied by 10, 0.1 s increments) high-density features for each experimental condition (300 spectra in total). By calculating both high- and low-density 2DFT, users are provided with a broad overview of the frequency content within the low-density scan, allowing the training of the computer for the gaits discrimination, and the additional high-density matrix provides a tool to calculate the extent of the deviations that the feature is exhibiting within one particular gait condition.

A training database for each participant and each sensor was created consisting of the high-density 2DFT (300 spectra in total, 100 spectra per condition, each containing 3 s-long extracts of the experimental conditions) to search the database for components that exhibited high variation and to allow their clustering. A simple machine learning algorithm consisting of principal component analysis (PCA) and discriminant function analysis (DFA) was executed on each training database. To run the PCA analysis for each experimental condition, PC scores (a set of scores transforming dependent variables of a multivariate database to a smaller set of principal components [40]) were attributed independently for each training database of each participant. PC scores were calculated to the 80th percentile for each variable, accounting for 80% of the total variance for each gait modality, participant, and sensor. As PC scores are ordered in decreasing variance, they differ from participant to participant as variance in each data set is not the same; hence, we chose a fraction of 80% of the total variance, meaning PC scores taken forward were not necessarily the same across each participant, sensor or condition.

Discriminant function analysis, also known as linear discriminant analysis, was used in two dimensions to further cluster the newly reduced data dimensionality by identifying discriminating features between the three experimental conditions. DFA is a supervised training method that achieves maximum discrimination between given conditions [40]. 

#### 2.4.2. Discrimination Quantification

**Figure 5:**
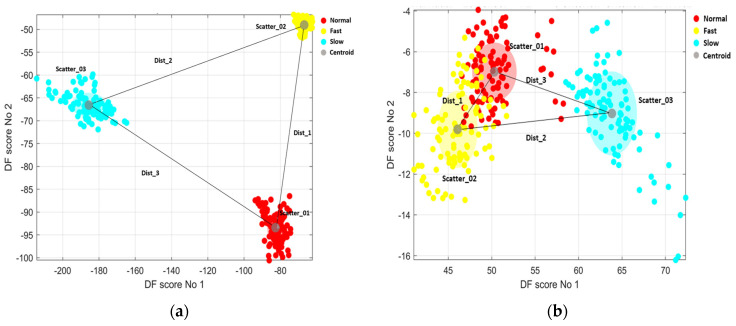
Discriminant function scores show three activities clustered separately. Criterion scores were further calculated using the following equation: Discrimination criterion = $\left(\frac{\mathrm{Dist}\_1\times \mathrm{Dist}\_2\times \mathrm{Dist}\_3}{\mathrm{Scatter}\_01\times \mathrm{Scatter}\_02\times \mathrm{Scatter}\_03}\right)$. (**a**) Shows successful discrimination with a criterion score larger than 500, cloud centroids exhibit large separation between one another, and the cluster standard deviations are relatively small. (**b**) Shows unsuccessful discrimination with a criterion score smaller than 500, two experimental condition cloud centroids in close proximity (normal and fast walking), and relatively large cluster standard deviations.

Once DFA was applied, DF scores 1 and 2 were plotted on the *X* and *Y* axes, respectively (Figure 4). The discrimination quality was then quantified and estimated by computing a discrimination criterion score, using a ratio of the product of the centroid distances between the three clusters to the product of the standard deviations of each cluster, as a large distance between centroids combined with a low standard deviation per condition represents strong discriminatory performance using DFA. The discrimination criterion was defined as follows:

$$\mathrm{Discrimination}\;\mathrm{criterion}=\left(\frac{Dist\_1\times Dist\_2\times Dist\_3}{Scatter\_01\times Scatter\_02\times Scatter\_03}\right),$$

where dist_1 is the distance between fast and normal cloud centroids, Dist_2 is the distance between fast and slow cloud centroids, and Dist_3 is the distance between normal and slow cloud centroids. Scatter_01, Scatter_02 and Scatter_03 are estimated by calculating standard deviations of the distance of each DF score to the centroid for each experimental condition (Figure 5).

To assess whether discrimination between the three locomotion modes was successful or unsuccessful, experimenters visually inspected the overlapping of the clouds for different experimental conditions. Severely overlapping conditions (Figure 5) resulted in the discrimination being deemed unsuccessful. Inspection of the clouds revealed that a discrimination criterion value of greater than 500 results in clouds exhibiting zero overlap between walking conditions. Clouds exhibit some overlap between conditions when the criterion score is smaller than 500; therefore, a success criterion threshold of 500 was selected. Successful and unsuccessful discrimination attempts were recorded for all five sensors (lower back, left and right thigh segments, and left and right shank segments), providing each sensor location with a total number of successful discriminations between the three experimental conditions.

## 3. Results

**Figure 6:**
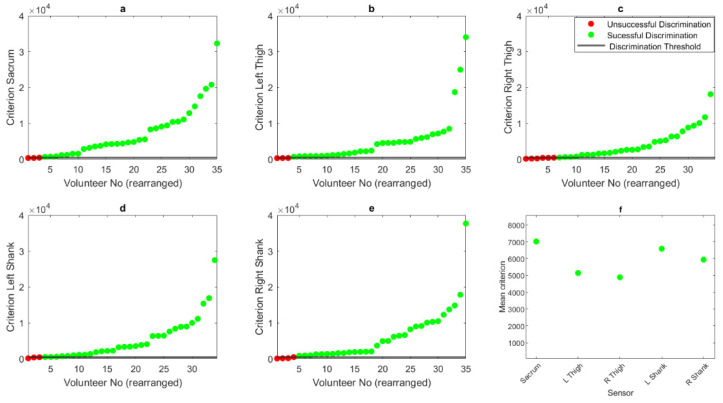
Criterion scores for all participants (rearranged left-right from lowest to highest) for (**a**) sacrum, (**b**) left thigh, (**c**) right thigh, (**d**) left shank and (**e**) right shank sensors. (**f**) Highlights mean criterion scores for all sensors.

The results show that the algorithm could successfully discriminate between locomotion types, with 91% of outcomes being successful in the sacrum, with a mean criterion of 7009, a combined success rate of 87% with a mean criterion score of 4943 in the left and right thigh segments and 90% success rate and mean criterion of 6212 in the shank segments (Figure 6). Figure 6 also highlights the individual criterion scores of each participant for each sensor location. Visual inspection highlights that the sacrum had more participants exhibiting a higher criterion value.

## 4. Discussion

The present study aimed to quantify the performance of a machine learning algorithm in the discrimination of three gait modalities: self-selected slow, self-selected normal, and self-selected fast walking, using data from wearable sensors attached to a range of commonly used physical attachment sites. Results showed that applying a combined unsupervised PCA and supervised DFA algorithm to a two-dimensional Fourier transform, alongside a ratio calculation, led to successfully quantifying the discrimination quality of self-selected slow, normal and fast gait modalities, with the sacrum and shanks exhibiting the strongest discrimination, followed by the thighs.

The successful application of the two-dimensional Fourier transform highlights that it is a valuable tool for gait analysis using accelerometer data when subtle changes to locomotor patterns are executed. The 2DFT enabled us to extract comprehensive frequency information from acceleration signals during normal, slow and fast walking conditions (Figure 3). When viewed as stacked arrays, the series of 2DFT showed apparent differences in gait frequencies, providing the machine learning tool with unique information on acceleration signals and allowing for successful discrimination. Previous work [39,41] using 2DFT to highlight the variation in frequency harmonics demonstrates that 2DFT is highly suited to discriminating between different spectral signatures [39]. Gait signatures in healthy populations provide the 2DFT with highly similar features with each step due to their cyclical nature. These repeatable features allow the 2DFT to successfully identify different locomotor activity patterns when either slowing or increasing their gait speed, resulting in successful discrimination when simple machine learning methods are applied. Being able to differentiate between gait speeds is important as gait speed is often used as a biomarker for health, more broadly health and wellbeing [42], pathological progression [43,44], and rehabilitation [45].

Machine learning algorithms can be used to identify activities of daily living. When using wearable sensors, the input is raw acceleration, and features are chosen for the algorithm to try and detect the activities. The algorithm used in this paper uses discriminant function plots and a ratio calculation to consistently report how well the different gait modalities have been discriminated. Knowledge of the quality of discrimination will aid future optimisation processes, providing users with a quantifiable method when optimising algorithmic parameters. 

Quantification can also allow clinicians to make informed decisions on the attachment location. Understanding that one attachment area yields greater activity discrimination quality will enable researchers to add to the field, potentially narrowing down on when or what causes discrimination failure, whether due to hardware error, location attachment point error, or an error related to the activity type and failure with recognition. If the issue was due to errors in discriminating between activity types, the discriminant function plots allow users to identify which activities are causing the discrimination to fail with a visual inspection of which clusters exhibit overlapping features on the scatterplots. 

The machine learning algorithm used in this study shows that one sensor is adequate for identifying cyclical locomotor tasks when placed on the lower extremity. Several previous studies have opted to fuse wearable sensor data, combining the data from multiple attachment sites to provide information on locomotion [29,30]. Although these methods have worked in classifying locomotor tasks, the fusion of numerous sensors makes data collection more costly due to the requirement of more sensors. It also adds unnecessary additional steps to the processing efforts. The present study shows that using one sensor at the sacrum or on the shanks can yield success rates greater than 90%, giving researchers and clinicians the scope to collect data at one location using one sensor. This may be particularly meaningful for research into patients with particular pathologies whereby individuals use lower limb assistive technology such as prosthetic or orthotic devices. Sensors could be placed on the devices on the lower extremity, minimising skin contact with the sensor and potentially increasing participant adherence, especially in longer-term data collection.

Another strength of the present study is that only triaxial accelerations are needed for data processing. Previous works aiming to identify or discriminate between activities have successfully used IMUs [4,13,14,15,16,18,22,25,26,27,28,32], which requires a greater computational load, leading to shorter battery life. Results from this study are particularly relevant to those who want to measure locomotion in a real-world setting for a prolonged period. As locomotion and gait speed is used as a marker for and can be used to inform prescribing assistive devices [46], knowledge of how an individual changes through locomotion types over several weeks has the potential to alter clinical practices and prescriptions. In addition, the ability to use one sensor in a minimum of three attachment locations allows clinicians and patients to work together to find an area that is the most comfortable for the user.

One limitation associated with the current study is that it does not explore the underlying mechanisms in cases where discrimination was not possible. Investigating causes for failure to discriminate could be performed in the future to further advance this methodological approach. Future work could also address the limitation that the current study used a strictly healthy cohort. Follow-up research would need to apply the methodology to pathological populations to assess whether the same results are seen. Additionally, future works could utilise the current algorithm to assess different real-world activities, such as running, slope walking or stair negotiation, adding to the depth of understanding of how people interact with their environment in the real world.

## 5. Conclusions

The present work presents a novel method of discriminating between locomotion types using a single accelerometer at different attachment locations. The algorithm combines data reduction, feature selection and classification methods to cluster three locomotor tasks. The study also quantifies the quality of the discrimination between clusters, allowing users to see what or where the algorithm works most successfully. The creation of a machine learning algorithm sensitive enough to identify signals as sensitive as different walking speeds opens up the ability to identify further cyclical activities of daily living. The ability to identify how well the algorithm discriminates between gait modalities allows future users to modify and seek to optimise the algorithm dependent on the research question.

## Data Availability

Data are contained within the article. The MATLAB code is openly available here: https://github.com/Liamdhughes/activity_discrimination_PCA_DFA. Our 2DFT and scatter plot images are available here: https://figshare.com/projects/Activity_recognition_accelerometer_project/179508.

## References

[1] Rehman R.Z.U., Del Din S., Shi J.Q., Galna B., Lord S., Yarnall A.J., Guan Y., Rochester L. (2019). Comparison of Walking Protocols and Gait Assessment Systems for Machine Learning-Based Classification of Parkinson’s Disease. Sensors.

[2] Buckley C., Alcock L., McArdle R., Rehman R., Del Din S., Mazzà C., Yarnall A., Rochester L. (2019). The Role of Movement Analysis in Diagnosing and Monitoring Neurodegenerative Conditions: Insights from Gait and Postural Control. Brain Sci..

[3] Rehman R.Z.U., Zhou Y., Del Din S., Alcock L., Hansen C., Guan Y., Hortobágyi T., Maetzler W., Rochester L., Lamoth C.J.C. (2020). Gait Analysis with Wearables Can Accurately Classify Fallers from Non-Fallers: A Step toward Better Management of Neurological Disorders. Sensors.

[4] Dauriac B., Bonnet X., Pillet H., Lavaste F. (2019). Estimation of the walking speed of individuals with transfemoral amputation from a single prosthetic shank-mounted IMU. Proc. Inst. Mech. Eng. H.

[5] Noh B., Yoon H., Youm C., Kim S., Lee M., Park H., Kim B., Choi H., Noh Y. (2021). Prediction of Decline in Global Cognitive Function Using Machine Learning with Feature Ranking of Gait and Physical Fitness Outcomes in Older Adults. Int. J. Environ. Res. Public Health.

[6] Trentzsch K., Schumann P., Śliwiński G., Bartscht P., Haase R., Schriefer D., Zink A., Heinke A., Jochim T., Malberg H. (2021). Using Machine Learning Algorithms for Identifying Gait Parameters Suitable to Evaluate Subtle Changes in Gait in People with Multiple Sclerosis. Brain Sci..

[7] Maki B.E. (1997). Gait Changes in Older Adults: Predictors of Falls or Indicators of Fear?. J. Am. Geriatr. Soc..

[8] Perera S., Patel K.V., Rosano C., Rubin S.M., Satterfield S., Harris T., Ensrud K., Orwoll E., Lee C.G., Chandler J.M. (2016). Gait Speed Predicts Incident Disability: A Pooled Analysis. J. Gerontol. Ser. A.

[9] Quach L., Galica A.M., Jones R.N., Procter-Gray E., Manor B., Hannan M.T., Lipsitz L.A. (2011). The Nonlinear Relationship Between Gait Speed and Falls: The Maintenance of Balance, Independent Living, Intellect, and Zest in the Elderly of Boston Study. J. Am. Geriatr. Soc..

[10] Rochat S., Büla C.J., Martin E., Seematter-Bagnoud L., Karmaniola A., Aminian K., Piot-Ziegler C., Santos-Eggimann B. (2010). What is the Relationship Between Fear of Falling and Gait in Well-Functioning Older Persons Aged 65 to 70 Years?. Arch. Phys. Med. Rehabil..

[11] Mason R., Pearson L.T., Barry G., Young F., Lennon O., Godfrey A., Stuart S. (2023). Wearables for Running Gait Analysis: A Systematic Review. Sports Med..

[12] Soltani A., Dejnabadi H., Savary M., Aminian K. (2020). Real-World Gait Speed Estimation Using Wrist Sensor: A Personalized Approach. IEEE J. Biomed. Health Inform..

[13] Dixon P.C., Schütte K.H., Vanwanseele B., Jacobs J.V., Dennerlein J.T., Schiffman J.M., Fournier P.-A., Hu B. (2019). Machine learning algorithms can classify outdoor terrain types during running using accelerometry data. Gait Posture.

[14] Mahoney J.M., Rhudy M.B. (2019). Methodology and validation for identifying gait type using machine learning on IMU data. J. Med. Eng. Technol..

[15] Chen W.-H., Lee Y.-S., Yang C.-J., Chang S.-Y., Shih Y., Sui J.-D., Chang T.-S., Shiang T.-Y. (2020). Determining motions with an IMU during level walking and slope and stair walking. J. Sports Sci..

[16] Beaufils B., Chazal F., Grelet M., Michel B. (2019). Robust Stride Detector from Ankle-Mounted Inertial Sensors for Pedestrian Navigation and Activity Recognition with Machine Learning Approaches. Sensors.

[17] Lee M.-W., Khan A.M., Kim J.-H., Cho Y.-S., Kim T.-S. A single tri-axial accelerometer-based real-time personal life log system capable of activity classification and exercise information generation. Proceedings of the 2010 Annual International Conference of the IEEE Engineering in Medicine and Biology.

[18] Li Z., Wei Z., Yue Y., Wang H., Jia W., Burke L.E., Baranowski T., Sun M. (2015). An Adaptive Hidden Markov Model for Activity Recognition Based on a Wearable Multi-Sensor Device. J. Med. Syst..

[19] Procter D.S., Page A.S., Cooper A.R., Nightingale C.M., Ram B., Rudnicka A.R., Whincup P.H., Clary C., Lewis D., Cummins S. (2018). An open-source tool to identify active travel from hip-worn accelerometer, GPS and GIS data. Int. J. Behav. Nutr. Phys. Act..

[20] Chu A.H.Y., Bernard J.Y., Koh D., Müller-Riemenschneider F. (2021). Accelerometer Profile of Physical Activity and Sedentary Behavior in a Multi-Ethnic Urban Asian Population. Res. Q. Exerc. Sport.

[21] Lonini L., Gupta A., Kording K., Jayaraman A. Activity recognition in patients with lower limb impairments: Do we need training data from each patient?. Proceedings of the 2016 38th Annual International Conference of the IEEE Engineering in Medicine and Biology Society (EMBC).

[22] Lonini L., Gupta A., Deems-Dluhy S., Hoppe-Ludwig S., Kording K., Jayaraman A. (2017). Activity Recognition in Individuals Walking with Assistive Devices: The Benefits of Device-Specific Models. JMIR Rehabil. Assist. Technol..

[23] Aziz O., Musngi M., Park E.J., Mori G., Robinovitch S.N. (2017). A comparison of accuracy of fall detection algorithms (threshold-based vs. machine learning) using waist-mounted tri-axial accelerometer signals from a comprehensive set of falls and non-fall trials. Med. Biol. Eng. Comput..

[24] Mannini A., Sabatini A.M. (2011). Accelerometry-Based Classification of Human Activities Using Markov Modeling. Comput. Intell. Neurosci..

[25] Mannini A., Sabatini A.M. (2014). Walking speed estimation using foot-mounted inertial sensors: Comparing machine learning and strap-down integration methods. Med. Eng. Phys..

[26] Benson L.C., Clermont C.A., Osis S.T., Kobsar D., Ferber R. (2018). Classifying running speed conditions using a single wearable sensor: Optimal segmentation and feature extraction methods. J. Biomech..

[27] Hu B., Dixon P.C., Jacobs J.V., Dennerlein J.T., Schiffman J.M. (2018). Machine learning algorithms based on signals from a single wearable inertial sensor can detect surface- and age-related differences in walking. J. Biomech..

[28] Skaramagkas V., Pentari A., Kefalopoulou Z., Tsiknakis M. (2023). Multi-Modal Deep Learning Diagnosis of Parkinson’s Disease—A Systematic Review. IEEE Trans. Neural Syst. Rehabil. Eng..

[29] McGinnis R.S., Mahadevan N., Moon Y., Seagers K., Sheth N., Wright J.A., DiCristofaro S., Silva I., Jortberg E., Ceruolo M. (2017). A machine learning approach for gait speed estimation using skin-mounted wearable sensors: From healthy controls to individuals with multiple sclerosis. PLoS ONE.

[30] Ejupi A., Galang C., Aziz O., Park E.J., Robinovitch S. Accuracy of a wavelet-based fall detection approach using an accelerometer and a barometric pressure sensor. Proceedings of the 2017 39th Annual International Conference of the IEEE Engineering in Medicine and Biology Society (EMBC).

[31] Aziz O., Klenk J., Schwickert L., Chiari L., Becker C., Park E.J., Mori G., Robinovitch S.N. (2017). Validation of accuracy of SVM-based fall detection system using real-world fall and non-fall datasets. PLoS ONE.

[32] Yurtman A., Barshan B. (2017). Activity Recognition Invariant to Sensor Orientation with Wearable Motion Sensors. Sensors.

[33] Ma Y., Ghasemzadeh H. An asynchronous multi-view learning approach for activity recognition using wearables. Proceedings of the 2016 38th Annual International Conference of the IEEE Engineering in Medicine and Biology Society (EMBC).

[34] Martindale C.F., Sprager S., Eskofier B.M. (2019). Hidden Markov Model-Based Smart Annotation for Benchmark Cyclic Activity Recognition Database Using Wearables. Sensors.

[35] Fukushi K., Huang C., Wang Z., Kajitani H., Nihey F., Nakahara K. (2022). On-Line Algorithms of Stride-Parameter Estimation for in-Shoe Motion-Sensor System. IEEE Sens. J..

[36] Figueiredo J., Santos C.P., Moreno J.C. (2018). Automatic recognition of gait patterns in human motor disorders using machine learning: A review. Med. Eng. Phys..

[37] Cleland I., Kikhia B., Nugent C., Boytsov A., Hallberg J., Synnes K., McClean S., Finlay D. (2013). Optimal Placement of Accelerometers for the Detection of Everyday Activities. Sensors.

[38] Ramsey M.-T. (2018). The Ethology of Honeybees (*Apis mellifera*) Studied Using Accelerometer Technology. Ph.D. Thesis.

[39] Hall H., Bencsik M., Newton M. (2023). Automated, non-invasive Varroa mite detection by vibrational measurements of gait combined with machine learning. Sci. Rep..

[40] Bisele M., Bencsik M., Lewis M.G.C., Barnett C.T. (2017). Optimisation of a machine learning algorithm in human locomotion using principal component and discriminant function analyses. PLoS ONE.

[41] Ramsey M.-T., Bencsik M., Newton M.I., Reyes M., Pioz M., Crauser D., Delso N.S., Le Conte Y. (2020). The prediction of swarming in honeybee colonies using vibrational spectra. Sci. Rep..

[42] Binotto M.A., Lenardt M.H., Rodríguez-Martínez M.d.C. (2018). Fragilidade física e velocidade da marcha em idosos da comunidade: Uma revisão sistemática. Rev. Esc. Enferm. USP.

[43] Stansfield B., Hawkins K., Adams S., Church D. (2018). Spatiotemporal and kinematic characteristics of gait initiation across a wide speed range. Gait Posture.

[44] Grande G., Triolo F., Nuara A., Welmer A.-K., Fratiglioni L., Vetrano D.L. (2019). Measuring gait speed to better identify prodromal dementia. Exp. Gerontol..

[45] Rose D.K., Nadeau S.E., Wu S.S., Tilson J.K., Dobkin B.H., Pei Q., Duncan P.W. (2017). Locomotor Training and Strength and Balance Exercises for Walking Recovery After Stroke: Response to Number of Training Sessions. Phys. Ther..

[46] Barnett C.T., Hughes L.D., Sullivan A.E., Strutzenberger G., Levick J.L., Bisele M., De Asha A.R. (2021). Exploring the interaction of knee and ankle component use on mobility test performance in people with unilateral transfemoral amputation. Prosthet. Orthot. Int..

